# Association between red blood cell transfusions and clinical outcome in critically ill children

**DOI:** 10.1186/cc12306

**Published:** 2013-03-19

**Authors:** P Demaret, M Tucci, T Ducruet, H Trottier, J Lacroix

**Affiliations:** 1 CHC - Clinique de l'Espérance, Montegnée, Belgium; 2 CHU Sainte-Justine, Montreal, Canada

## Introduction

Red blood cell (RBC) transfusions are frequent in critically ill children. Their benefits are clear in several situations. However, issues surrounding their safety have emerged in the past decades. It is important to identify the potential complications associated with RBC transfusions, in order to evaluate their risk-benefit ratio better.

## Methods

A single-center prospective observational study of all children admitted to the pediatric intensive care unit (PICU) over a 1-year period. The variables possibly related to RBC transfusions were identified before the study was initiated, and their presence was assessed daily for each child. In transfused cases (TCs), a variable was considered as a possible outcome related to the transfusion only if it was observed after the first transfusion.

## Results

During the study period, 913 admissions were documented, 843 of which were included in the study. Among them, 144 (17%) were transfused. When comparing TCs with nontransfused cases (NTCs), the odds ratio (OR) of new or progressive multiple organ dysfunction syndrome (NPMODS) was 2.39 (95% CI = 1.58 to 3.62, *P <*0.001). This association remained statistically significant in the multivariate analysis for children with admission PRISM score ≤5 (OR = 2.41, 95% CI = 1.08 to 5.37, *P *= 0.032). TCs were ventilated longer than NTCs (14.1 ± 32.6 days vs. 4.3 ± 9.6 days, *P <*0.001). This difference was still significant after adjustment using a Cox model. Moreover, we observed an adjusted dose-effect relationship between RBC transfusions and length of mechanical ventilation. The PICU length of stay was significantly increased for TCs (12.4 ± 26.2 days vs. 4.9 ± 10.2 days, *P <*0.001), even after multivariate adjustment (hazard ratio of PICU discharge for TCs: 0.61, 95% CI = 0.5 to 0.74, *P <*0.001). We also observed an adjusted dose-effect relationship between RBC transfusions and PICU length of stay. The paired analysis for comparison of pre-transfusion and post-transfusion values showed that the arterial partial pressure in oxygen was significantly reduced after the first transfusion (mean difference

42.8 mmHg, 95% CI = 27.2 to 58.3, *P <*0.001). The paired analysis also showed an increased proportion of renal replacement therapy, while the proportions of sepsis, severe sepsis and septic shock did not differ.

## Conclusion

RBC transfusions were associated with prolonged mechanical ventilation and prolonged PICU stay. The risk of NPMODS was increased in some transfused children. Moreover, our study questions the ability of stored RBCs to improve oxygenation in critically ill children. These results should help to improve transfusion practice in the PICU.

